# Transport of Perfluoroalkyl Substances (PFAS) by Three Renal Transporters: Implications for PFAS Bioaccumulation Mechanisms

**DOI:** 10.1021/envhealth.6c00088

**Published:** 2026-06-20

**Authors:** Shan Niu, Yifei Ma, Arundhati Tewari, Carla Ng

**Affiliations:** † Advanced Interdisciplinary Institute of Environment and Ecology, Guangdong Provincial Key Laboratory of Wastewater Information Analysis and Early Warning, School of Technology for Sustainability, Beijing Normal University, Zhuhai 519087, China; ‡ Department of Civil & Environmental Engineering, 6614 University of Pittsburgh, Pittsburgh, Pennsylvania 15261, United States

**Keywords:** OAT1, P-glycoprotein, breast cancer resistance protein, efflux transporters, PFAS replacements

## Abstract

The long biological half-lives of per- and polyfluoroalkyl substances (PFAS) in humans have been linked to interactions with renal transport proteins and polypeptides. However, only a limited number of kidney transporters have been studied for their ability to transport specific PFAS. Moreover, few studies have investigated whether PFAS alternatives serve as substrates for these transporters. In this study, we focused on one renal influx transporter, organic anion transporter 1 (OAT1) and two efflux transporters (P-glycoprotein (P-g) and breast cancer resistance protein (BCRP)) involved in PFAS excretion. We evaluated two well-studied PFAS, perfluorooctanoic acid (PFOA) and perfluorooctanesulfonate (PFOS), along with two current substitutes. Hexafluoropropylene oxide dimer acid (HFPO-DA), the major component of the PFOA alternative GenX, was not transported by any of the studied renal transporters. In contrast, hexafluoropropylene oxide trimer acid (HFPO-TA), a minor component of GenX, as well as PFOA itself, were taken up by all studied transporters. Both PFOS and its tested alternative (F53B), including both major and minor components, were substrates of all tested renal transporters, with PFOS and F53B showing relatively higher transport activity. BCRP- and P-gp-mediated transport of five PFAS underwent a saturable process with Michaelis constant (*K*_m_) ranging from 1.29 to 7.92 μmol/L and 1.41 to 2.78 μmol/L, respectively. The highest *V*_max_/*K*_m_ ratio was observed for PFOA transport by P-gp (279 pmol/mg protein/min/(μmol/L)). To the best of our knowledge, this is the first study reporting interactions between renal transporters and these legacy PFAS replacements. These findings enhance our understanding of PFAS bioaccumulation at the cellular and molecular levels, offering key parameters for PFAS toxicokinetic modeling.

## Graphical abstract

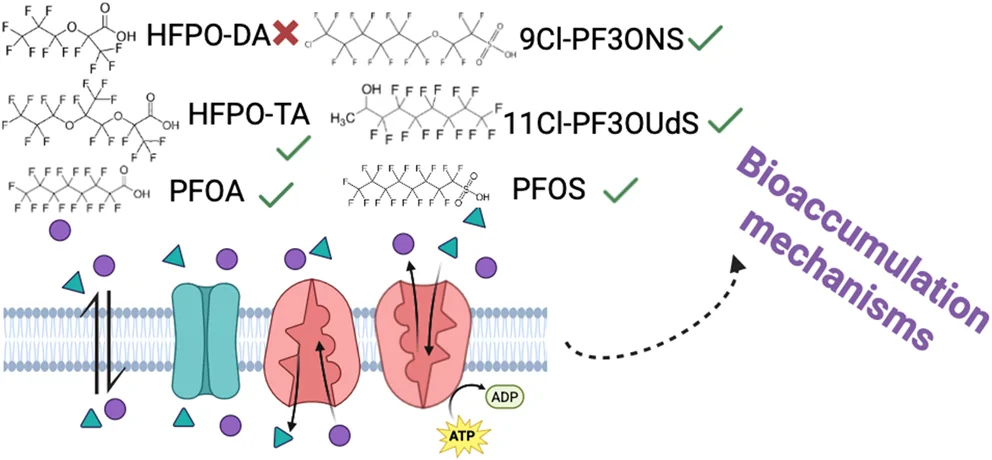

## Introduction

The adverse health impacts of per- and polyfluoroalkyl substances (PFAS) have caused increasing global attention. PFAS are a class of manufactured chemicals with varied structures containing at least one perfluorinated methyl or methylene carbon. Given their unique chemical properties, PFAS have been widely used in various consumer and industrial products, leading to their ubiquity in the environment^1^ and human blood.^2^ Exposure to perfluorooctanoic acid (PFOA) and perfluorooctanesulfonate (PFOS), two well-studied PFAS, is associated with various adverse health outcomes, including but not limited to kidney disease,^3^ liver disease,^4^ and altered immune function.^5^ The half-lives of PFOA and PFOS in humans range from 2–3 and 3–4 years, respectively,^6,7^ indicating that they can persist in the body for years, amplifying health concerns. Due to their significant health risks, PFOS and PFOA were phased out of production and use in the United States in 2002 and 2015, respectively.^8^ As a result, several alternatives that are themselves PFAS, including short-chain perfluoroalkyl acids (with fewer than six fluorinated carbon atoms) and ether-based PFAS, have emerged. Notably, GenX (a trade name) containing hexafluoropropylene oxide dimer acid (HFPO-DA) as a major component and hexafluoropropylene oxide trimer acid (HFPO-TA) as a minor component has been developed and used as a replacement for PFOA in fluoropolymer manufacture.^9,10^ In addition, F53B (a trade name) with potassium 9-chlorohexadecafluoro-3-oxanonane-1-sulfonate (9Cl-PF3ONS) as a major component and 11-chloroeicosafluoro-3-oxaundecane-1-sulfonate (11Cl-PF3OUdS) as a minor component has been used as an alternative to PFOS, primarily in China. Unfortunately, these alternatives have been shown to be toxic, bioaccumulative, or both, raising concerns about their continued use and environmental release.^11,12^

Protein binding and associations with membrane phospholipids have been shown to be two important mechanisms underlying PFAS bioaccumulation.^13^ Furthermore, studies have shown that the long half-lives of PFOA and PFOS are linked to the activity of kidney transport proteins and polypeptides.^14,15^ The involvement of several renal transporters in facilitating PFAS transport has been confirmed. For instance, human organic anion transporters 1 and 3 (OAT1 and OAT3) were confirmed to transport PFOA, thereby facilitating its uptake into the kidneys.^16,17^ Additionally, human OAT4,^16^ urate transporter 1 (URAT1),^18^ and rat Oatp 1a1 ^19^ have all demonstrated the capability to transport PFOA from urine to the kidney, contributing to its reabsorption, which may be one of the reasons for the long biological half-life of PFOA. Furthermore, human OATP2B1 ^20^ and apical sodium-dependent bile acid transporter (ASBT)^21^ also have the ability to transport PFOS from urine to the kidney, while the bidirectional transporter OSTα/β aids in PFOS transport from the blood to the kidney.^21^ Our previous state-of-the-science review identified that efflux transporters, those responsible for transporting substrates out of the kidneys either to the blood or urine, are largely untested for PFAS transport.^14^ In addition, seven untested kidney transporters, including three known efflux transporters, have been proposed to have the potential for PFAS transport based on structural similarities between PFAS and their substrate molecules.^14^ The suggested three known efflux transporters are multidrug resistance protein 1 (MRP1), P-glycoprotein (P-gp), and breast cancer resistance protein (BCRP).^14^ Beyond the gap in knowledge regarding renal transporters, few replacement PFAS have been tested as transporter substrates.

To enhance the understanding of renal transporter-mediated PFAS transport, we focused on three renal transporters, one uptake transporter (OAT1) and two efflux transporters (P-gp and BCRP), examining their ability to transport PFOA, PFOS, and their ether-based replacements (HFPO-DA, HFPO-TA, 9Cl-PF3ONS, and 11Cl-PF3OUdS). The findings of this study provide insights into PFAS bioaccumulation at the cellular and molecular levels, offering key parameters for PFAS toxicokinetic modeling.

## Methods

### Materials

PFOA, HFPO-DA, HFPO-TA, and PFOS were purchased from SynQuest Laboratories (Alachua, FL, USA), and 9Cl-PF3ONS and 11Cl-PF3OUdS (50 μg/mL) were purchased from Wellington Laboratories (Guelph, Ontario, CA). Human embryo kidney (HEK) 293 cells (GM1101G), control vesicles (GM0003), human P-gp vesicles (GM0015), human BCRP vesicles (GM0008), cell culture medium, and uptake solutions were all obtained from ThermoFisher (Waltham, MA, USA). The ORF of OAT1 in the vector pcDNA (SLC22A6, Accession No. NM_004790.5) and the vector control with enhanced green fluorescent protein insert were purchased from GenScript (Piscataway, NJ, USA).

### Cell Culture and Transporter Expression

HEK293 cells were maintained in Dulbecco’s Modified Eagle Medium (DMEM), supplemented with 10% fetal bovine serum (FBS) and 100 U/mL penicillin/streptomycin at 37 °C in a humidified 5% CO_2_ atmosphere. HEK293 cells were transfected with OAT1 plasmid DNAs or vector control as mock cells using Lipofectamine 2000 (ThermoFisher, Waltham, MA, USA) according to the manufacturer’s instructions. At 48 h post-transfection, selection was initiated by supplementing the culture medium with 450 μg/mL G418 sulfate (ThermoFisher, Waltham, MA, USA). Stable resistant clones were subsequently obtained under the selection conditions.

### Transport Assay and Kinetic Analysis

OAT1-mediated uptake was assessed in OAT1-transfected HEK293 cells, whereas P-gp and BCRP efflux activities were evaluated using membrane vesicles with mixed orientation, in which only the inside-out vesicle fraction supports ATP-dependent transport. This approach reflects the distinct directional functions of these transporters, as whole-cell assays are suitable for influx transporters and vesicle-based assays allow direct measurement of ATP-dependent efflux. This strategy is consistent with recommended practices outlined by the International Transporter Consortium.^21^ For OAT1 transport, HEK293 cells were seeded on poly-d-lysine-coated 24-well plates 48 h before the start of the uptake assay. Stock solutions of PFAS were prepared in methanol and diluted to desired concentrations using an uptake buffer (Hank’s balanced salt solution (HBSS) containing 20 mmol/L HEPES). Both the pH and methanol content of all final transport solutions were adjusted to 7.4 and ≤1%, respectively, to ensure they had no effect on the cell function. To start the uptake assay, cells were washed three times with 500 μL of 37 °C prewarmed uptake buffer. Substrate uptake was initiated by adding 500 μL of 37 °C prewarmed dosing solution that contained the desired concentration of PFAS. Preliminary experiments indicated that the 10 s incubation time was technically challenging to perform reproducibly. By contrast, the 30 s incubation time was achieved reliably and reproducibly and was therefore included in the study. Cells were incubated at 37 °C in a humidified 5% CO_2_ atmosphere for the designed incubation times: 30 s, 1, 1.5, 2, 2.5, 5, and 10 min. These time points were initially tested until 30 min to make sure they covered the linear uptake phase until equilibrium was reached. Uptake was terminated by the removal of the dosing solution and washing of the cells three times with 500 μL of ice-cold uptake buffer. For the time-dependent kinetics experiment, a 10 μmol/L exposure concentration was used for PFOA, HFPO-DA, HFPO-TA, and PFOS. A concentration of 10 μmol/L was suitable for achieving equilibrium or kinetic measurements and was commonly used in previous studies of transporter interactions with PFAS.^16,17,22^ Due to substantially lower solubility, a 4 μmol/L concentration was used for 9Cl-PF3ONS and 11Cl-PF3OUdS. All kinetics experiments were run for 10 min, after which cells were lysed in 300 μL of 1% Triton X-100 in DI water held at room temperature for 20 min. 25 μL of the resulting cell lysate was used to measure the protein concentration. After centrifuging at 20,000*g* for 10 min, 50 μL of the remaining cell lysate was used for PFAS measurements. HEK293 mock cells were treated the same way as OAT1-expressing HEK293 cells and served as negative controls.

For the vesicle-based uptake assay, the protocol was based on the manufacturer’s instructions with modifications. Specifically, 15 μL of 2 mg/mL transporter vesicles or control vesicles were added to a 96-well plate, which was placed on ice. 15 μL of reaction buffer (ThermoFisher, Waltham, MA, USA) and 30 μL of test compound solution (prepared using reaction buffer with 3-fold desired concentrations) were subsequently added to each well. Uptake was started by adding 30 μL of MgATP solution and incubating the plate with shaking at 37 °C for the designed time. Control vesicles without transporter expression were included in the vesicle uptake experiment as negative control to account for background uptake. Thus, transporter-associated uptake was evaluated by comparison between transporter vesicles and control vesicles processed in parallel under the same assay conditions. Uptake was terminated by adding 45 μL of ice-cold stop solution (ThermoFisher, Waltham, MA, USA). The assay mixture was then centrifuged at 9000*g* for 10 min to pellet the vesicles, and the supernatant was carefully removed without disturbing the pellet. To reduce potential bias from nonspecific binding and remove residual external PFAS, the vesicle pellet was washed twice with ice-cold stop solution by gentle resuspension, with each wash followed by centrifugation and removal of the wash supernatant. The washed vesicle pellet was then subjected to PFAS extraction and quantification. Based on the protein content in vesicles relative to that in HEK293 cells, a concentration of 1 μmol/L was used for the six tested PFAS. Uptake capabilities were normalized to protein content, which accounted for differences in uptake concentrations. In addition, our preliminary data up to 10 min indicated that pseudoequilibrium was reached at or before 2.5 min in vesicle assays, consistent with previous observations for perfluorobutanesulfonic acid, perfluorohexanesulfonic acid, and PFOS across several human transporters.^20^ Therefore, uptake experiments were performed at 30 s, 1, 1.5, 2, and 2.5 min. All uptake assays, including the HEK293-based and vesicle-based assays, were conducted in triplicate.

### Protein and PFAS Measurement

The total protein concentrations were determined using a bicinchoninic acid (BCA) protein assay (ThermoFisher, Waltham, MA, USA). PFAS concentrations were measured using our previously developed analytical method with ultrahigh-performance liquid chromatography coupled with a triple quadrupole mass spectrometer (UHPLC-MS/MS; Vanquish Flex and TSQ Quantis, Thermo Scientific).^23^ A delay column (C18 2.6 μm, 50 mm × 4.6 mm; Thermo Scientific) was used to prevent potential instrumental in-source PFAS contamination. Separation was performed on a C18 column (1.7 μm, 50 mm × 2.1 mm; Waters Acquity UPLC). Mobile phases A and B consisted of 20 mM ammonium acetate and methanol, respectively, at a flow rate of 0.25 mL/min. The gradient profile was as follows: 5% B at the start, increasing to 50% over 3 min, 80% over 11 min, 95% over 14 min, and then returning to the initial 5% over 1 min, held for 3 min. Mass spectrometry used the multiple reaction monitoring (MRM) mode, and parameters were adapted from Niu et al.^24^ Electrospray ionization (ESI) in negative ionization mode was employed, with settings at 2.5 kV for spray voltage, 325 °C for the ion transfer tube temperature, and 300 °C for the vaporizer temperature.

For HEK cell-based samples, 50 μL of centrifuged cell lysate was transferred to a polypropylene LC vial and mixed with 50 μL of methanol. For vesicle-based samples, 90 μL of the centrifuged solution was transferred to a polypropylene LC vial and mixed with 10 μL of methanol. After vortexing for 15 s, the samples were analyzed by UHPLC-MS/MS for PFAS quantification.

### Statistical Analysis

The experiments were performed in triplicate for each HEK293 and vesicle-based uptake assays. Data are expressed as means and standard deviations of the mean (specific data for the plots can be found in the Supporting Information). Statistical significance was analyzed using a two-tailed paired *t* test. Data were considered statistically significant at *p* < 0.05.

## Results and Discussion

### OAT1-Mediated Transport of PFAS

The uptake of six selected PFAS by human OAT1-transfected HEK293 cells (labeled as OAT1) and wild-type HEK293 cells (labeled as HEK293) is presented in Figure 1. The uptake was conducted at 10 μmol/L for PFOA, HFPO-DA, HFPO-TA, and PFOS individually for 10 min and 4 μmol/L for 9Cl-PF3ONS and 11Cl-PF3OUdS for 10 min because of their relatively lower solubility in the buffer solution. Addition of chlorine atoms to the fluorinated chain imparts higher hydrophobicity and may also confer higher bioaccumulation potential.^25,26^ Significant net OAT1-mediated uptake was observed for PFOA, HFPO-TA, PFOS, 9Cl-PF3ONS, and 11Cl-PF3OUdS but not for HFPO-DA, which was confirmed as not being transported in both time-dependent and concentration-dependent kinetic experiments. This is not surprising given the high water solubility of HFPO-DA. Previous studies have also found that HFPO-DA is not bioaccumulative.^27–29^ This study indicates that all tested compounds except HFPO-DA are OAT1 substrates, with uptake rates in the order of 9Cl-PF3ONS > PFOS > 11Cl-PF3OUdS > PFOA > HFPO-TA (Figure 1). Human OAT1 was previously tested for PFOA transport,^16,17,22^ and our results (net uptake of 150 ± 40 pmol/mg protein at 10 μmol/L measured at 1 min; data shown in Figure 2a) are within the reported range of ∼100 to ∼200 pmol/mg protein at 10 μmol/L for 1 min.^17,22^ To the best of our knowledge, this study is the first to determine OAT1 transport for HFPO-TA, PFOS, 9Cl-PF3ONS, and 11Cl-PF3OUdS, as well as the first report for OAT1 *not* facilitating the transport of HFPO-DA. To further understand species differences, we compared our results with previous studies that reported PFAS transport by rat Oat1. An inferential human population study conducted by Ducatman et al.^30^ also suggested that human OATs have a lower capability for PFAS excretion compared to rat Oats. However, this pattern was not observed in Nakagawa et al.’s study,^17^ which reported a similar capacity for PFOA uptake between human OAT1 and rat Oat1. Further investigation is needed to better understand observed species differences in PFAS disposition and their underlying mechanisms.

**Figure 1:**
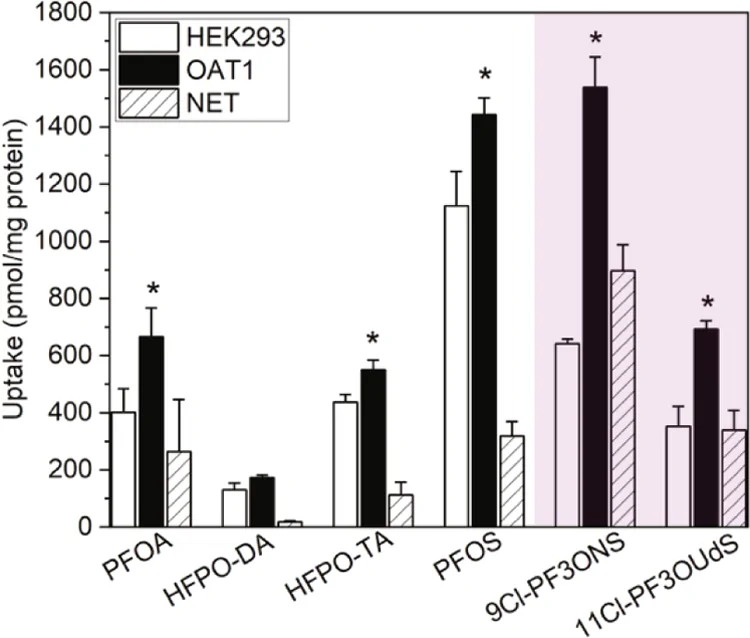
Uptake of six tested PFAS by wild-type HEK293 cells (empty bar), human OAT1-transfected HEK293 cells (solid bar), and net uptake (bar with diagonal lines). Each bar represents mean ± SD of triplicates. The asterisk (*) in the figure indicates a significant difference in PFAS uptake between wild-type HEK293 cells and human OAT1-transfected HEK293 cells. Note that uptake experiments were performed with PFOA, HFPO-DA, HFPO-TA, and PFOS at 10 μmol/L for 10 min, while for 9Cl-PF3ONS and 11Cl-PF3OUdS (indicated by light purple shading), the concentration was 4 μmol/L for 10 min due to their low solubilities in water.

**Figure 2:**
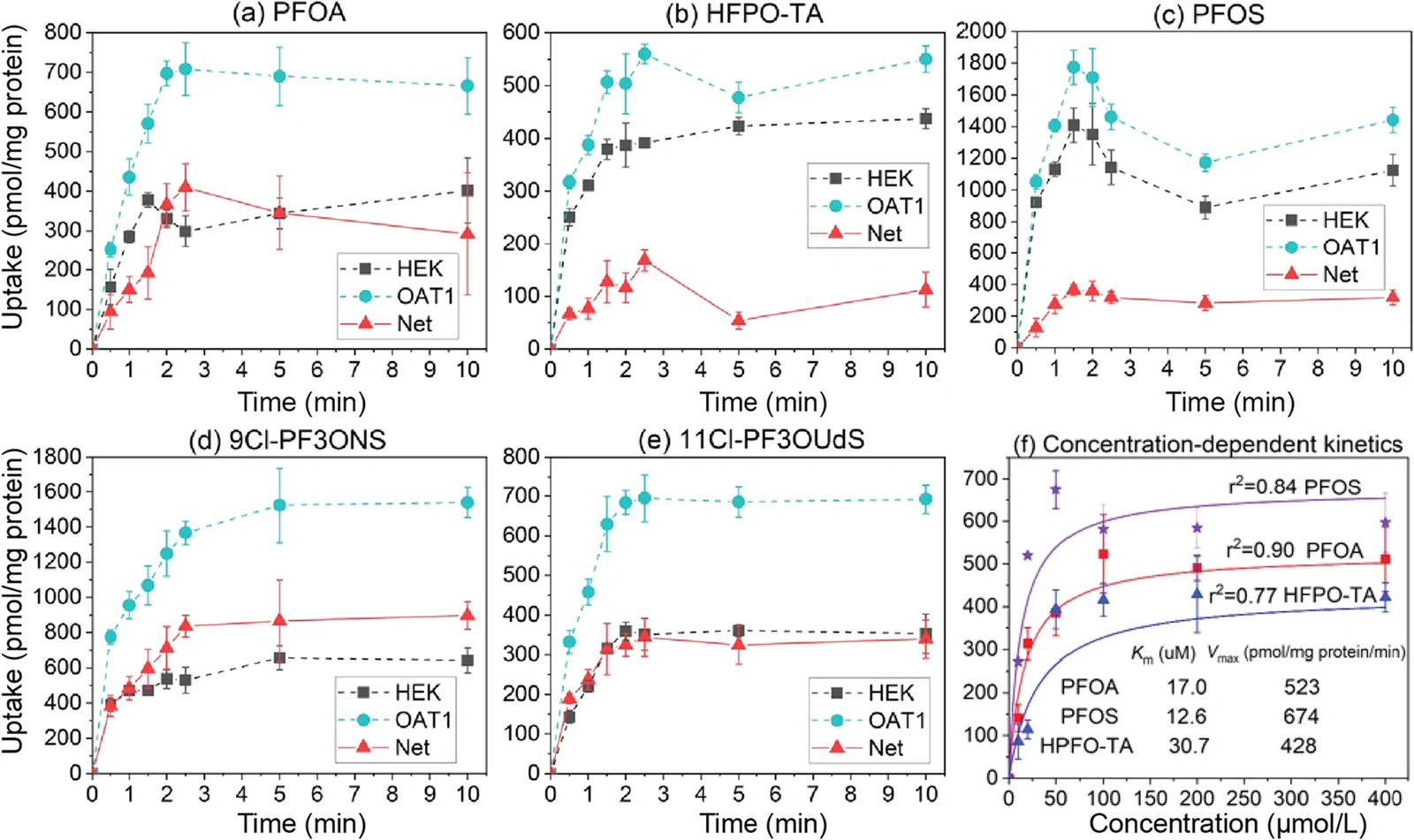
(a–e) Time-dependent kinetics of PFAS transport by wild-type HEK293 cells (black rectangles), human OAT1-transfected HEK293 cells (teal circles), and net human OAT1-mediated PFAS transport (red triangles). Net uptake was obtained by subtracting the uptake values for wild-type cells from OAT1-transfected cells. (f) Concentration-dependent net uptakes of PFAS at 1 min. The net concentration-dependent OAT1-mediated uptake was fitted to the Michaelis–Menten equation. Each point represents mean ± SD of triplicates.

The observed uptake of PFAS by wild-type HEK293 cells could be achieved in two ways: passive diffusion (including via phospholipid membrane binding) and transporter-mediated uptake by other transporters present at background levels (i.e., not intentionally overexpressed) in the cells. Concentrations of 9Cl-PF3ONS and 11Cl-PF3OUdS were both 4 μmol/L, and those for other chemicals were at 10 μmol/L for the uptake experiment. Among the chemicals at 10 μmol/L, PFOS showed the highest uptake concentration by wild-type HEK293 cells, followed by PFOA ≈ HFPO-TA > HFPO-DA. Notably, despite the concentrations being 4 μmol/L, both 9Cl-PF3ONS and 11Cl-PF3OUdS demonstrated higher concentrations in wild-type HEK293 compared to PFOA, HFPO-TA, and HFPO-DA, indicating higher accumulation potentials.

Time- and concentration-dependent kinetic studies were conducted to further understand PFAS transport by human OAT1. Linear uptake by human OAT1 was observed until 1.5–2 min for all tested PFAS. Note that HFPO-DA was excluded from this section given that human OAT1 did not transport HFPO-DA. Additionally, 9Cl-PF3ONS and 11Cl-PF3OUdS were excluded from the concentration-dependent assays due to their low solubilities, which precluded higher concentration dosing in buffer. The concentration-dependent net OAT1-mediated uptake rates were fitted to the Michaelis–Menten equation, and the *K*_m_ (the Michaelis constant, which is the substrate concentration at which the reaction velocity is 50% of the maximum) and *V*_max_ (the maximum velocity achieved by the system at maximum (saturating) substrate concentrations) values are listed in Figure 2f. Some variability and fluctuations were observed in the experimental data, e.g., in the time-course and kinetic analyses. The fitted kinetic parameters are intended to describe the overall uptake trend rather than exact values. A lower *K*_m_ indicates higher affinity, while a higher *V*_max_ means greater transporting activity. The affinity of studied PFAS to human OAT1 follows this order: PFOS > PFOA > HFPO-TA, with the transport velocities following the same order. In addition, the *V*_max_/*K*_m_ ratio, an indicator of intrinsic clearance and overall transport efficiency, was determined to quantify OAT1-mediated transport of the studied PFAS. Among the tested compounds, OAT1 exhibited the highest transport efficiency for PFOS (53.5 pmol/mg protein/min/(μmol/L)), followed by PFOA (30.8 pmol/mg protein/min/(μmol/L)) and HFPO-TA (13.9 pmol/mg protein/min/(μmol/L)), suggesting that PFOS has a higher affinity for OAT1 and/or is transported more efficiently compared to the other PFAS.

### P-gp and BCRP-Mediated Transport of PFAS

P-gp and BCRP are two efflux transporters located at the apical membrane of human proximal tubular cells. The ability of these transporters to facilitate the transport of PFAS aids in the excretion of PFAS from renal cells into urine, thereby promoting PFAS elimination. Evaluation of PFAS uptake by two human efflux transporters, namely, P-gp and BCRP, was conducted at a concentration of 1 μmol/L for each chemical for 2.5 min using vesicular transport assays. Similar to human OAT1, both P-gp and BCRP showed the capacity to transport PFOA, HFPO-TA, PFOS, 9Cl-PF3ONS, and 11Cl-PF3OUdS. At 2.5 min, the capability of P-gp and BCRP for PFAS transport followed similar trends: 11Cl-PF3OUdS > PFOA > HFPO-TA ≈ 9Cl-PF3NS > PFOS (Figure 3). In addition, neither P-gp nor BCRP demonstrated transport capabilities for HFPO-DA (data not shown). Therefore, HFPO-DA was excluded from this section. Time-dependent kinetics were conducted at a concentration of 1 μmol/L for each chemical except for 9Cl-PF3ONS and 11Cl-PF3OUdS, which were excluded from concentration-dependent experiments due to their low solubility in buffer. Linear uptake was observed within 1–2 min for both P-gp and BCRP for the five studied PFAS. The *K*_m_ and *V*_max_ for P-gp and BCRP are presented in Figure 3b,d. The half-maximal constants (*K*_m_) for P-gp followed the order PFOA > PFOS ≈ HFPO-TA, while the maximum transport velocities (*V*_max_) followed the order: HFPO-TA > PFOS > PFOA. On the other hand, *K*_m_ for BCRP followed PFOA > HFPO-TA > PFOS, with *V*_max_ following HFPO-TA > PFOA > PFOS.

**Figure 3:**
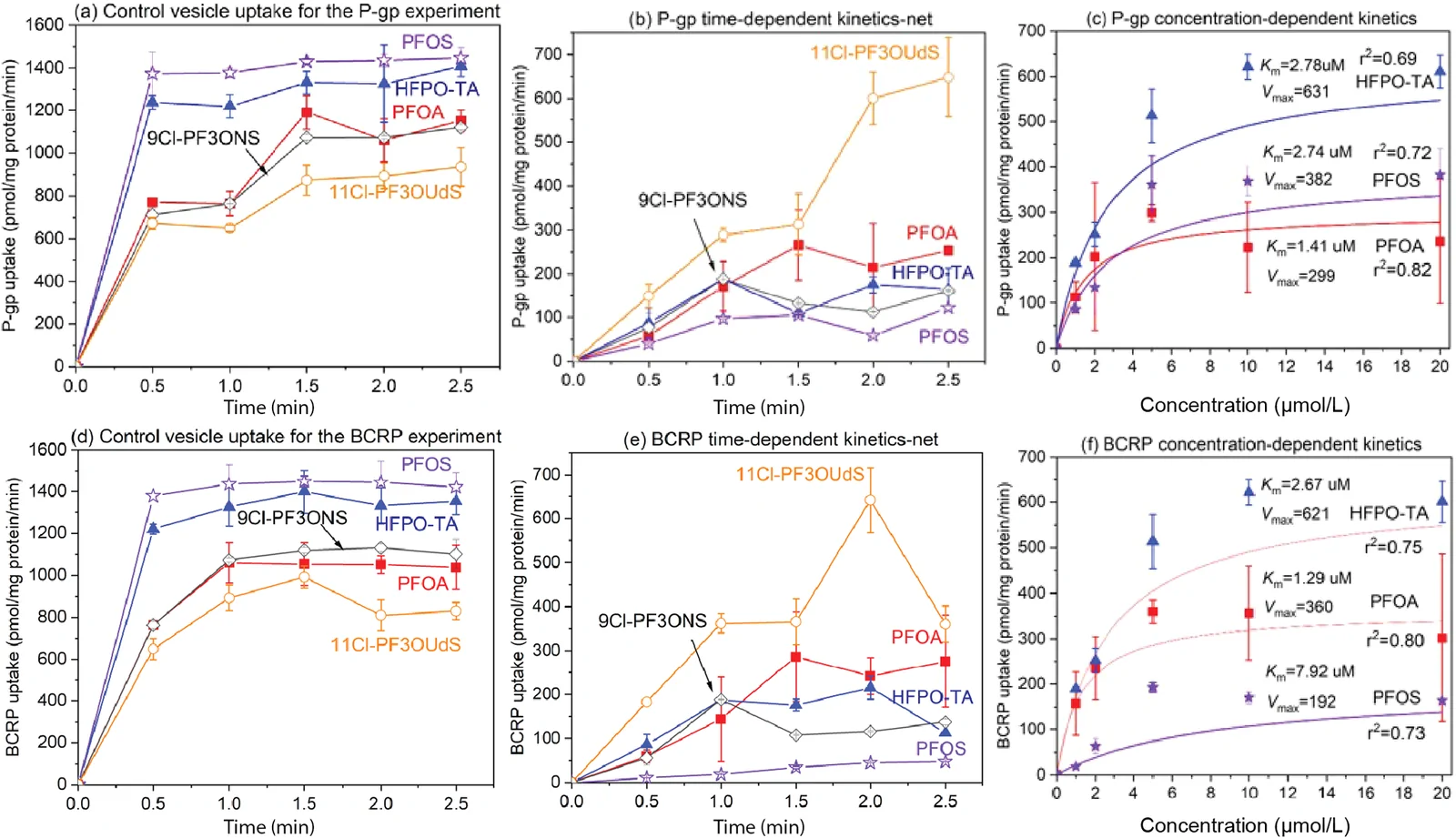
Control and net P-gp uptakes of PFAS for (a, b) time- and (c) concentration-dependent kinetics at 1 min. Control and net BCRP uptakes for PFAS for (d, e) time- and (f) concentration-dependent kinetics at 1 min. The net uptake was obtained by subtracting uptakes in control vesicles from transporter vesicles. Each point represents mean ± SD of triplicates. All points are plotted with error bars; some are too small to see.

The highest ratio of *V*_max_/*K*_m_ of P-gp was observed for PFOA (279 pmol/mg protein/min/(μmol/L)), followed by HFPO-TA (234 pmol/mg protein/min/μmol/L) and PFOS (24 pmol/mg protein/min/(μmol/L)). In addition, the *V*_max_/*K*_m_ ratios for BCRP followed the order: HFPO-TA (227 pmol/mg protein/min/(μmol/L)) > PFOA (212 pmol/mg protein/min/(μmol/L)) > PFOS (139 pmol/mg protein/min/(μmol/L)). Detailed investigation of the mechanisms underlying transporter–PFAS interactions is needed to further understand and generalize the potential for PFAS transport by renal transporters, particularly in relation to the apparent affinity of these transporters for specific PFAS as reflected by *K*_m_ values.

The determination of uptake by vesicles was achieved by assessing the differences in concentrations before and after the designed experiments. Since PFAS might absorb onto the plate wall, it was rinsed with methanol after the experiment. We found less than 1% of PFAS was absorbed, which had a negligible influence on the results. Relatively high uptake of PFAS was observed in control vesicles (1000–2000 pmol/mg protein) compared to wild-type HEK293 cells (100–400 pmol/mg protein). This difference might be due to distinctions in the biological structure between vesicles and cells. Vesicles have a high surface area-to-volume ratio, facilitating passive diffusion, while HEK293 cells are larger with a lower surface-area-to-volume ratio and featuring membrane asymmetry and lipid rafts, which may limit PFAS diffusion efficiency.^31,32^ These differences may contribute to the higher background PFAS observed in control vesicles compared with HEK293 cells.

Because of variations in chemical concentrations used in the *in vitro* experiments for OAT1 compared to those for P-gp and BCRP, the specific rates of PFAS uptake cannot be directly compared between these transporters. However, the ability of each transporter to transport different PFAS can still be assessed under their respective experimental conditions. Notably, OAT1, P-gp, and BCRP showed different orders in the calculated kinetic parameters (*K*_m_ and *V*_max_) for the studied PFAS.

## Conclusions

### Implications for Bioaccumulation

Comparison of equilibrium transport capabilities showed higher PFOS uptake by the influx transporter OAT1 (318 pmol/mg protein at 10 min) compared to the efflux transporters P-gp (121 pmol/mg protein at 2.5 min) and BCRP (48 pmol/mg protein at 2.5 min). In addition, the protein level measured in kidney cortex was higher for OAT1 (5.3 ± 1.9 pmol/mg) than that of P-gp (2.1 ± 0.8 pmol/mg) and BCRP (below the detection limit/much lower than the OAT1 protein level) in humans (*n* = 20).^33^ These suggest that OAT influx transporters might contribute substantially to PFOS bioaccumulation and prolonged half-lives, while efflux transporters may have limited capacity for excretion. A similar pattern was also observed for PFOA transport, although the difference among transporters was relatively smaller. This may partially explain why the biological half-life of PFOS is longer than PFOA. Overall, our results provide supporting evidence that the long half-lives of both PFOS and PFOA may be associated with renal transporter activity. For 9Cl-PF3ONS, higher uptake capabilities were observed via OAT1 (896 pmol/mg protein) compared to the efflux transporters P-gp (161 pmol/mg protein) and BCRP (137 pmol/mg protein). These values suggest that 9Cl-PF3ONS may have a high bioaccumulation potential or long half-life, potentially even greater than that of PFOS. This is consistent with previous studies that report the half-life of 9Cl-PF3ONS to be as long as 18.5 years.^34^ In contrast, a different uptake pattern (P-gp > BCRP > OAT1) was observed for 11Cl-PF3OUdS; however, the reason is not yet clear. Interestingly, yet not surprisingly, HFPO-DA was not a substrate for any of the transporters studied, supporting observations that this chemical is not bioaccumulative. A previous study predicted the half-life of HFPO-DA to be 3.4 days.^35^ In contrast, HFPO-TA was a substrate for all the transporters studied, but the uptake capabilities were similar across transporters, indicating that it may be bioaccumulative, though its half-life is likely not as prolonged as that of PFOA.

Membrane transporters play an important role in the mediation of PFAS entry and efflux from cells. Moreover, these transporters can be expressed in multiple tissues and serve different purposes (uptake vs elimination). For example, OAT1 is not only present in the kidney but also in the brain, endocrine tissue, and gastrointestinal tract. Similarly, P-gp and BCRP are widely distributed, including in the eye, lung, kidney, gastrointestinal tract, liver, skin, and adipose. Furthermore, specific transporters exhibit sex-specific expression patterns, potentially leading to differences in PFAS toxicokinetics. The ability of certain uptake transporters (e.g., OAT1) to facilitate the transport of certain PFAS can therefore contribute to PFAS bioaccumulation in multiple tissues. Conversely, specific efflux transporters (e.g., P-gp and BCRP) can aid in the PFAS elimination. This study provides useful *in vitro* information on transporter-mediated kinetics (e.g., *V*_max_ and *K*_m_), which may contribute to the understanding of *in vivo* dynamics; however, the latter are influenced by additional and more complex physiological factors. A suite of transporters, including both influx and efflux types, may have the capability to transport PFAS. Only three transporters were measured in this study, which is not sufficient to fully characterize the impact of transporters on PFAS bioaccumulation. We acknowledge that variability in the experimental data results in some discrepancies among individual data points and the fitted curves. These deviations likely reflect inherent experimental uncertainty and biological variability, and therefore, the fitted kinetic parameters should be interpreted as approximations of overall uptake trends rather than precise quantitative values. Given the extensive diversity across transporter families and PFAS, more studies, both experimental and computational, are needed to fully understand the roles and mechanisms of PFAS transport by membrane transporters, which underlie the toxicokinetics of PFAS. In addition, while cell-based studies are valuable for elucidating specific transport mechanisms, their predictive power for whole-organism outcomes remains limited because of the complex interplay between uptake and efflux processes across different tissues. Physiologically based toxicokinetic (PBTK) modeling incorporating transporter mechanisms offers a valuable framework for understanding the *in vivo* bioaccumulation and kinetics of PFAS. For example, studies by Cheng et al.^13^ and Niu et al.^14^ have shown that the OAT1-mediated transport directly prolongs the half-life of PFOA in rats. More comprehensive incorporation of transporter pathways, particularly efflux transporters, is needed in both PBTK models and the experimental investigation of PFAS bioaccumulation dynamics.

## Supplementary Material



## Notes

Tables containing all uptake data used to construct Figures 1–3 (PDF)
