# Supplementary material for: Transport of Perfluoroalkyl Substances (PFAS) by Three Renal Transporters: Implications for PFAS Bioaccumulation Mechanisms
Source: Environ Health (Wash). 2026 Jun 20;4(9):2150–7. doi: 10.1021/envhealth.6c00088 (PMC13595387; doi:10.1021/envhealth.6c00088)
Supplement: Supplementary file 1 [file eh6c00088_si_001.pdf]

Supporting Information for:

Transport of Perfluoroalkyl Substances (PFAS) by Three Renal Transporters: Implications for PFAS Bioaccumulation Mechanisms

Shan Niu,[1,2*] Yifei Ma,[2] Arundhati Tewari,[2] Carla Ng[2*]

[1]Advanced Interdisciplinary Institute of Environment and Ecology, Guangdong Provincial Key Laboratory of Wastewater Information Analysis and Early Warning, School of Technology for Sustainability, Beijing Normal University, Zhuhai 519087, China

[2]Department of Civil & Environmental Engineering, University of Pittsburgh, Pittsburgh, PA 15261, USA.

*Co-corresponding authors

Email address: Shan Niu shan.niu@bnu.edu.cn

Carla Ng carla.ng@pitt.edu

Table of Contents:

Table 1: data used for Figure 1……………………………………………………………page S2
Table 2: data used for Figure 2……………………………………………………………page S3
Table 3: data used for Figure 3 for P-gP………………………………………………...page S5
Table 4: data used for Figure 3 for BCRP………………………………………………page S6

Specific data used for the plots

Table 1. Uptake (pmol/mg protein) of six tested PFAS by wild-type HEK293 cells, human OAT1-transfected HEK293 cells, and net uptake (data used for Figure 1).

Chemicals	HEK 293	OAT1	Net uptake
**PFOA**	402	610	208
	484	621	137
	320	766	446
**HFPO-DA**	109	182	73
	129	172	43
	154	166	12
**HFPO-TA**	421	523	102
	464	543	79
	427	584	157
**PFOS**	1244	1502	258
	1128	1497	369
	1001	1329	328
**9Cl-PF3ONS**	658	1645	987
	628	1536	908
	641	1435	794
**11Cl-PF3OUdS**	422	722	300
	330	641	311
	307	715	408

Table 2: Uptake (pmol/mg protein) of five tested PFAS by wild-type HEK293 cells, human OAT1-transfected HEK293 cells, and net uptake (data used for Figure 2).

Chemicals	Time (min)	HEK 293		OAT1		Net uptake	
		Average	SD	Average	SD	Average	SD
**PFOA**	0	0	/	0	/	0	/
	0.5	157	44.6	251	19.0	93.9	43.6
	1	285	15.6	435	46.0	150	33.0
	1.5	378	18.1	571	48.8	192	65.7
	2	331	22.6	697	31.5	366	53.5
	2.5	298	40.0	708	66.3	410	59.4
	5	345	38.8	690	73.6	345	93.1
	10	402	82.2	666	71.4	292	154
**HFPO-DA**	0	0	/	0	/	0	/
	0.5	251	16.2	318	12.6	67.2	11.2
	1	311	9.4	388	18.6	77.1	20.0
	1.5	379	19.3	507	21.0	127	40.1
	2	387	41.7	503	56.7	116	28.3
	2.5	392	6.4	560	18.1	168	20.1
	5	423	16.4	477	29.0	54.4	16.0
	10	437	19.0	550	25.4	113	32.9
**HFPO-TA**	0	0	/	0	/	0	/
	0.5	924	27.9	1050	45.6	127	59.1
	1	1132	46.5	1406	40.0	274	59.7
	1.5	1408	108.4	1774	109	365	35.0
	2	1351	194.2	1709	182	358	62.2
	2.5	1143	108.9	1460	81.2	317	37.7
	5	890	72.0	1172	55.0	283	46.9
	10	1124	99.2	1443	80.4	318	46.1
**PFOS**	0	0	/	0	/	0	/
	0.5	391	36.9	775	40.4	384	60.3
	1	471	27.4	955	78.0	484	65.5
	1.5	472	16.9	1068	110	596	109
	2	538	55.1	1249	128	711	122
	2.5	530	74.0	1366	66.9	836	61.4
	5	657	68.2	1523	213	866	232
	10	642	72.3	1539	85.5	897	78.8
**9Cl-PF3ONS**	0	0	/	0	/	0	/
	0.5	143	16.3	332	29.0	189	12.8
	1	221	14.8	458	33.2	237	25.8
	1.5	315	10.6	629	69.6	314	64.8
	2	359	22.9	684	30.3	325	28.7
	2.5	351	40.2	695	59.6	344	48.0
	5	361	15.8	685	38.3	324	48.3
	10	353	49.5	693	36.4	340	48.3

**11Cl-PF3OUdS**	0	0	/	0	/	0	/
	0.5	157	44.6	251	19.0	94.3	43.6
	1	285	15.6	435	46.0	150	33.0
	1.5	378	18.1	571	48.8	192	65.7
	2	331	22.6	697	31.5	366	53.5
	2.5	298	40.0	708	66.3	410	59.4
	5	345	38.8	690	73.6	345	93.1
	10	402	82.2	666	71.4	292	154

Table 3: Uptake (pmol/mg protein) of five tested PFAS by control vesicles and human P-gp net uptake (P-gp uptake minus uptake in control vesicles) (data used for Figure 3 for P-gP).

**Chemicals**	**Time (min)**	**Control vesicle**		**P-gp net uptake**	
		**Average**	**SD**	**Average**	**SD**
**PFOA**	0	0	/	0	/
	0.5	770	17	57.5	1.7
	1	764	57	171	57
	1.5	1191	80	265	80
	2	1060	101	214	101
	2.5	1151	50	253	5.0
**HFPO-DA**	0	0	/	0	/
	0.5	1238	34	87.0	34
	1	1220	56	188	5.6
	1.5	1333	53	109	5.3
	2	1326	182	175	18.
	2.5	1408	48	164	48
**HFPO-TA**	0	0	/	0	/
	0.5	1373	101	40.2	1.0
	1	1378	22	97.1	2.2
	1.5	1430	11	105	1.2
	2	1435	29	58.6	2.9
	2.5	1446	48	122	4.8
**PFOS**	0	0	/	0	/
	0.5	711	32	76.8	32
	1	764	1.9	188	0.2
	1.5	1073	14	132	1.4
	2	1073	7.3	112	0.7
	2.5	1120	13	162	1.3
**9Cl-PF3ONS**	0	0	/	0	/
	0.5	672	27	149	27
	1	649	15	289	15
	1.5	873	69	313	69
	2	893	60	600	60
	2.5	935	90	648	90
**11Cl-PF3OUdS**	0	0	/	0	/
	0.5	770	17	57.5	1.7
	1	764	57	171	57
	1.5	1191	8	265	80
	2	1060	101	214	101
	2.5	1151	50	253	5.0

Table 4: Uptake (pmol/mg protein) of five tested PFAS by control vesicles and human BCRP net uptake (P-gp uptake minus uptake in control vesicles) (data used for Figure 3 for BCRP).

Chemicals	Time (min)	Control vesicle		BCRP net uptake	
		Average	SD	Average	SD
PFOA	0	0	/	0	/
	0.5	764	14	59.5	14
	1	1060	96	144	96
	1.5	1054	102	286	102
	2	1051	42	242	42
	2.5	1039	105	276	105
HFPO-DA	0	0	/	0	/
	0.5	1220	23	86.1	23
	1	1326	92	187	2
	1.5	1399	104	175	14
	2	1332	86	214	26
	2.5	1352	63	113	13
HFPO-TA	0	0	/	0	/
	0.5	1378	80	10.6	1
	1	1435	94	19.0	0.7
	1.5	1448	28	34.6	3
	2	1444	102	45.1	2
	2.5	1420	70	47.7	1
PFOS	0	0	/	0	/
	0.5	764	17	56.0	17
	1	1073	9	188	1.1
	1.5	1120	13	107	1.4
	2	1132	18	115	2.1
	2.5	1101	70	137	7.3
9Cl-PF3ONS	0	0	/	0	/
	0.5	649	50	183	14
	1	893	62	362	22
	1.5	993	52	365	52
	2	810	74	642	74
	2.5	830	41	360	41
11Cl-PF3OUdS	0	0		0.0	
	0.5	764	14	59.5	14
	1	1060	96	144	96
	1.5	1054	102	286	102
	2	1051	42	242	42
	2.5	1039	105	276	105
